# Breastfeeding Shapes the Gut Microbiota and Its Structure Is Associated with Weight Gain Trajectories in Mexican Infants

**DOI:** 10.3390/nu17050826

**Published:** 2025-02-27

**Authors:** Alejandra Arguelles-Lopez, Sandra V. Aguayo-Patrón, Ana M. Calderón de la Barca

**Affiliations:** Coordinación de Nutrición, Centro de Investigación en Alimentación y Desarrollo A.C., Hermosillo 83304, Mexico; aarguelles221@estudiantes.ciad.mx (A.A.-L.); sandra.aguayo@ciad.mx (S.V.A.-P.)

**Keywords:** breastfeeding, human milk composition, gut microbiota, infant growth

## Abstract

**Background:** Rapid weight gain in early infancy increases the risk of childhood obesity, while exclusive breastfeeding can protect against it, depending on breastmilk composition, maternal diet, and infant gut microbiota. **Objective:** The objective of this study was to analyze the association between maternal diet, breastmilk components, infant gut microbiota, and weight gain in the first year of life of Mexican breastfed infants. **Methods:** This longitudinal study included 27 mothers with exclusively breastfed infants (≥5 months of age). We evaluated maternal diet and breastmilk composition at 5 months postpartum (pp), the infant fecal microbiota at 5 and 12 months pp using 16S rRNA gene sequencing, and weight gain as normal, rapid or slow weight gain (NWG, RWG or SWG) in periods 1 (0–5.5 months) and 2 (5.5–12 months). **Results:** Infants with NWG in periods 1 and 2 made up 51% and 56%, respectively. In period 1, ingested breastmilk protein content was higher for NWG infants than for infants with SWG (*p* = 0.01), and the protein content was negatively correlated with maternal BMI (*r* = −0.42, *p* = 0.02). The genera *Veillonella* (19.5%), *Bifidobacterium* (19.5%), and *Escherichia-Shigella* (16.8%) dominated the microbiota at 5 months. At 12 months, *Bacteroides* predominated, and the first two genera remained. Breastmilk fat correlated with *Veillonella* abundance (*r* = −0.50, *p* = 0.02) and oligosaccharides with Lachnospiraceae (*r* = 0.73, *p* = 0.03) at 5 months. There was a trend of a higher abundance of *Bifidobacterium* in NWG infants than in other infants in period 1, while infants with RWG and SWG had a higher abundance of *Ruminococcus gnavus* (*p* = 0.03) in period 1 and *Alistipes* in period 2 (*p* = 0.01), respectively. **Conclusions:** Breastfeeding shaped the gut microbiota of exclusively breastfed infants, and its structure was associated with infant weight gain trajectories.

## 1. Introduction

The problem of excessive body weight and its associated health consequences has overwhelmed health systems worldwide. To address this problem in children and adults, it is essential to prioritize prevention in the first years of life which determines future health according to the DOHaD theory. As such, rapid weight gain trajectories during infancy have been associated with an increased risk of overweight and obesity in childhood [1]. Also, there is evidence to support the notion that breastfeeding, in terms of exclusivity and duration, may protect against childhood obesity [2,3].

Initially, the hypothesis that breastfeeding protects against obesity was inferred from data on the increase in body weight and the reduced prevalence of breastfeeding in different populations in the last decades. Recently, some authors have found that even infants who were exclusively breastfed for six months had a risk of developing childhood obesity if they presented rapid weight gain in the first months of life [4,5]. In contrast, a study involving 7074 Dane infants revealed that none of the infants who experienced rapid weight gain were overweight at age 5–9 years if they were exclusively breastfed for at least four months [2].

To explain the controversial results of the breastfeeding regime and the risk of excessive weight gain, additional information is needed regarding the mother’s conditions, including nutritional status and diet, breast milk composition, and environmental factors that may be implicated. In the last decades, a new factor has been involved: infant gut microbiota, which influences the body composition directly or indirectly, depending on its structure. Given that the gut microbiota is established in the first years of life, it is essential to investigate the specific mechanisms through which this relationship is established [6]. A preliminary step in this direction involves characterizing the microbiota composition in breastfeeding infants in different populations worldwide to identify common mechanisms of action across a range of microbiota structures.

Differences in the content of breast milk hormones could alter an infant’s eating behaviors, inducing alterations in the gut microbiota and body weight [7]. A prevailing view is that a high Firmicutes/Bacteroidetes ratio contributes to obesity [8]. In addition, the genera associated with anti-obesogenic properties following pathways such as polysaccharide degradation and essential amino acid synthesis are negatively associated with children’s weight [9], as are different genera that produce short-chain fatty acids [8]. A significant challenge is the wide variation in the gut microbiota composition of lactating infants; even in a small cohort, the abundance of phyla such as Actinobacteria and Firmicutes ranges from 0.5% to 76% [10]. Therefore, the genera and species combinations related to infant weight gain and risk of future obesity may be multiple.

There is a recently published study of infant weight gain and feeding regimes in a Mexican population from Central Mexico [4]. Our study population in Sonora, a state in Northwest Mexico, has particular characteristics, such as a very low population density of 16.7 inhabitants/km^2^ due to arid desert conditions that prevail in the region. The population is a homogeneous mestizo, with predominantly European ancestry and a low Amerindian component [11]. Furthermore, the prevalence of overweight and obesity in school-aged children is among the highest in the country (>39%) [12]. Finally, Sonora has one of the lowest prevalence of exclusive breastfeeding for at least 5 months considering that exclusive breastfeeding prevalence in Mexico is 34.2% [13]. Therefore, the northwestern Mexican population merits investigation of the relationship between maternal diet, breast milk composition, breastfeeding regime, weight gain, and the gut microbiota in the first year of life, which is the aim of the present study.

## 2. Materials and Methods

This longitudinal study included dyads of healthy mothers and their exclusively breastfed infants aged ≥5 months. Convenience sampling was conducted using the snowball method in Hermosillo, Sonora, in Northwest Mexico. Mothers were invited to participate through breastfeeding support groups on social media. The study was conducted from April 2022 to April 2024.

Inclusion criteria were as follows: mothers over 18 years of age who had a singleton pregnancy and prenatal follow-up. Mothers who suffered adverse conditions during pregnancy, such as preeclampsia or gestational diabetes, were excluded from the study, as were those with type 2 diabetes, infectious or immune diseases, or those who had been under pharmacological treatment or received antibiotics during lactation, or used drugs, alcohol, or smoked. Infants born preterm, those with a low birth weight, those receiving antibiotic treatment, those with conditions that could interfere with normal growth, or those who were introduced to other foods, aside from breast milk, before 5 months of age were excluded from the study. All participating mothers signed an informed consent form, and the study protocol was approved by the Ethics Committee of our institution (CEI/002/2022).

### 2.1. Anthropometric Measurements

During home visits, sociodemographic and health data were collected using questionnaires. Maternal weight and height were measured at 5.5 months postpartum using an electronic scale (FG-150KBM, A&D Company, Ltd., Tokyo, Japan) and a portable stadiometer (SECA 213, SECA, Hamburg, Germany). Body mass index (BMI) was calculated: a BMI < 18.5 was classified as underweight, 18.5–24.5 as normal weight, 25.0–29.9 as overweight, and ≥30.0 as obese. Infant weight and length were measured at 5.5 and 12 months of age using an electronic pediatric scale (SECA 334, SECA, Hamburg, Germany) and an infantometer (SECA 210, SECA, Hamburg, Germany). Mothers provided information on pre-gestational weight, weight gained during pregnancy, and the infant’s birth weight and length.

Infants’ weight-for-length Z-scores (WLZ) were obtained using the software Anthro v. 3.2.2 of the World Health Organization [14]. With these data, conditional weight gain was estimated, which is the residual of a linear regression of the WLZ at a given time of age and the previous WLZ, stratified by sex [15]. Conditional weight gain assesses the deviation from the infant’s expected weight based on a previous measurement and the growth of other infants in the same cohort [16]. It was assessed in two periods: 0 to 5.5 months (period 1) and 5.5 to 12 months (period 2). A conditional weight gain of <−0.67 was considered slow weight gain (SWG), −0.67 to 0.67 normal weight gain (NWG), and >0.67 rapid weight gain (RWG) [17].

### 2.2. Maternal Diet

The mothers’ dietary intake was evaluated at 5 months postpartum using a semi-quantitative food frequency questionnaire developed by Quizán-Plata et al. (2000) for Sonoran women. The FFQ was modified by adding foods that are nowadays commonly consumed and were not 20 years ago [18]. The dietary data were coded and analyzed using Ortega et al. and the Food Data Central databases [19,20]. The dietary intake was energy adjusted using the residual method. The dietary assessment included energy and the percentage of daily energy intake provided by proteins, carbohydrates, and fat. The mothers’ dietary intake data were part of a larger cohort that was previously published [21].

### 2.3. Breast Milk Composition

A breastmilk sample (10 mL approx.) at 5 months postpartum was obtained; it was mid-milk, collected between 9:00 and 11:00 a.m. The samples were collected using a manual pump, and stored at −70 °C. The protein in milk was quantified by the Lowry assay [22]. Reducing sugars in milk were analyzed by the phenol–sulfuric acid method [23]. Fat in milk was estimated using the crematocrit method [24].

For lactose quantification, after de-proteinization and derivatization, the milk samples (300 µL) were diluted with 12 mL of an ethanol:water mixture (80:20), and shaken overnight. Each supernatant was filtered using a 0.22 mm pore size nylon filter, and 20 mL were injected into a high-performance liquid chromatography (HPLC) system (Dionex Ultimate 3000, Thermo Scientific, San Jose, CA, USA). It was equipped with a pump (Accela 600, Thermo Scientific) and a refractive index detector (RefractoMax 520, Thermo Scientific), using a Microsorb 100-3 NH2 100 × 4.6 mm column for carbohydrates (Agilent Technologies, Santa Clara, CA, USA), an isocratic method with acetonitrile/water (80:20) as the mobile phase at a flow rate of 1.0 mL min^−1^. Lactose was quantified using external standards.

Total human milk oligosaccharides (HMOs) were estimated by subtracting the lactose concentration from the total reducing sugar concentration.

### 2.4. Microbiota Analysis

Infant stool samples were obtained at 5 and 12 months of age from soiled diapers. The samples were transported on ice to the lab. Each sample was homogenized, and an aliquot was stored at −60 °C for DNA extraction. DNA extraction was performed with the QIAmp Fast DNA Stool Mini Kit (QIAGEN, Hilden, Germany) following the manufacturer’s protocol. The quantity and quality of the extracted DNA were evaluated in a spectrophotometer Nanodrop 2000 (Thermo Scientific, Pittsburg, PA, USA).

Microbiota analysis was conducted by amplification of the V4 region of the 16S rRNA gene with the primers 515f-GTGBCAGCMGCCGCGGTAA and 806r-GGACTACCAGGGTATCTAAT [25]. Amplicons were purified and barcoded according to the Library Preparation user’s guide from Illumina. All the libraries, in equal concentrations, were mixed and sequenced in an Illumina Miniseq system (San Diego, CA, USA). The conditions were 300 cycles, with 2 × 150 pair-end format. Sequencing analysis was performed at the Microbial Genomics Lab in the Research Center for Food and Development (CIAD) in Mazatlán, Mexico.

### 2.5. Bioinformatics and Statistical Analysis

Data are presented as the mean and standard deviation for continuous variables, or sums (percentage) for categorical variables. The Shapiro–Wilk test was used to verify variable normality. Natural logarithmic transformation was applied to normalize variables with an abnormal distribution, such as HMOs and the microbiota taxa abundances. Pearson’s correlation test was used for all correlation analyses. Comparisons between two groups were made with the Mann–Whitney U test. Comparisons between three groups were made with an ANOVA when normality and equal variances were achieved; the Aspin–Welch test was used when normal data but unequal variances were found; and the Kruskal–Wallis test was employed for abnormal data. Significance was set at *p* < 0.05. All analyses were conducted using NCSS 2021 software. Prism GraphPad v. 9 was used for creating graphs.

The bioinformatic analysis started with the sequence cleaning (adapters and primers removing) and merging with the script pair-end_cleaner.sh developed by the Microbial Genomics Lab (CIAD, Mazatlán, Mexico; https://github.com/GenomicaMicrob/pair-end_cleaner (accessed 6 January 2025)). Once merged, sequences were imported into QIIME 2 v. 2024.5, and were de-noised and filtered with the DADA2 plugin, with a truncation length of 260 pb and a Phred score ≥ 20. The taxonomic assignation was made using the SKLEARN classifier and the human Stool Weighted Silva database (https://resources.qiime2.org/ accessed 8 January 2025). ASVs (amplicon sequence variants) were assigned with a 99% similarity threshold. Relative abundances were analyzed at the phylum and genus levels. The comparative analysis between the groups included only genera with >1% abundance.

Alpha and beta diversity were also analyzed in QIIME 2. The Shannon and Chao1 diversity indices were estimated to analyze alpha diversity. Meanwhile, beta diversity was evaluated employing weighted UniFrac distances and principal coordinate analysis of Bray–Curtis dissimilarities.

## 3. Results

**Figure 1:**
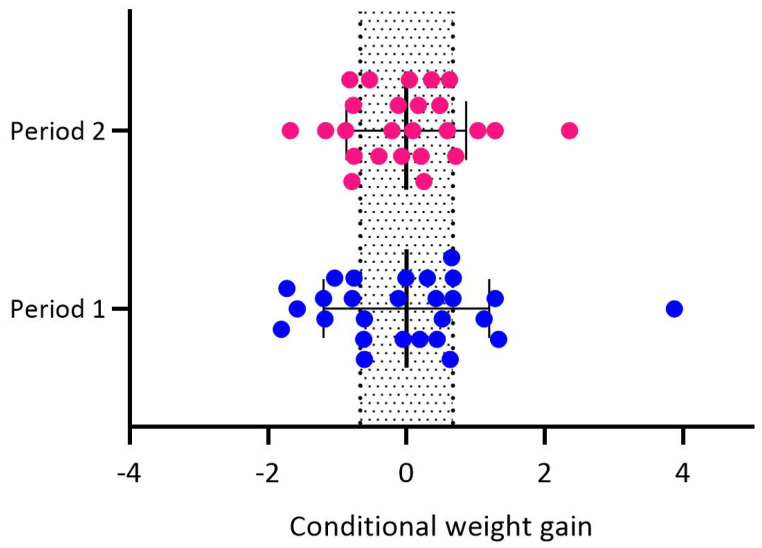
Infant weight gain was measured as conditional weight gain in periods 1 (0 to 5.5 months) and 2 (5.5 to 12 months). The dotted bar represents normal weight gain (−0.67 to 0.67).

We included 27 mother–infant dyads at baseline (5 months postpartum). At the follow-up (12 months postpartum), there were 25 mother–infant dyads. Table 1 presents maternal and infant general and perinatal characteristics. Regarding infant weight gain, 29.6% presented slow weight gain (SWG), 51.9% (NWG) and 18.5% had rapid weight gain (RWG) in period 1 (n = 27). In period 2 (n = 25), 28% experienced SWG, 56% NWG, and 16% RWG (Figure 1).

Fifty-two percent of mothers presented excess weight; therefore, we sought to analyze if maternal weight status was associated with infant growth. However, no significant correlation was found between maternal BMI, pre-gestational and at 5 months postpartum, and infant conditional weight gain in both periods (data not presented).

### 3.1. Maternal Diet and Breast Milk Composition

The daily energy intake of the mothers was 1853 ± 450 kcal, 47.4 ± 4.6 percent of which came from carbohydrates, 34.9 ± 4.2 percent from fat, and 17.6 ± 2.3 percent from proteins.

Table 2 presents breast milk composition at 5 months postpartum. There was no significant correlation between maternal diet and breast milk composition (data not presented). Maternal pre-gestational BMI was negatively correlated with protein concentration in breast milk at 5 months postpartum (*p* = 0.02) (Table 3). Additionally, infants with NWG in period 1 consumed breast milk with a higher protein concentration at 5 months of age than infants who experienced SWG (*p* = 0.01) (Table 4).

### 3.2. Infant Fecal Microbiota and Growth

The fecal microbiota of the infants enrolled in the study were analyzed at 5 (n = 26) and 12 months (n = 24). All samples met the quality criteria and were included in the analysis. Initially, 1,434,401 raw paired-end reads were obtained, and after quality filtering and de-noising, 960,928 reads were retained, resulting in an average of 19,218 reads per sample.

The microbial diversity in general was similar among the infant growth categories in study periods 1 and 2. There were no differences in alpha diversity, Shannon index, and Chao index (Figure S1a,b), nor in beta diversity, represented as weighted UniFrac distances (Figure S1c). Additionally, in the principal component analysis of Bray–Curtis dissimilarities, the samples from the three infant growth categories in period 1 were dispersed and overlapped. In period 2, the samples from the RGW were grouped (n = 3) but overlapped with the samples from the SWG and NWG groups that were more dispersed (Figure S1d,e).

**Figure 2:**
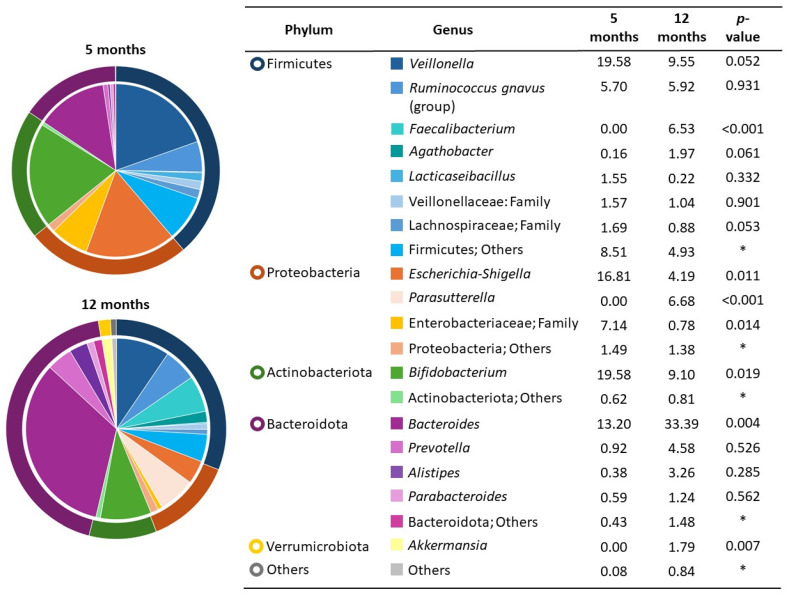
Infant fecal microbiota at phyla and genera levels at 5 months (n = 26) and 12 months of age (n = 24). The phyla relative abundances are in the rings around the pie charts and the genus relative abundances are presented in the pie fractions. Differences between the two groups were assessed with the Mann–Whitney U test. * Not calculated because of grouped unspecific data (multiple genera with abundance < 1%).

The taxonomic analysis revealed that at 5 months of age, the microbiota was dominated by the genera *Veillonella* (19.5%), *Bifidobacterium* (19.5%), and *Escherichia–Shigella* (16.8%). At 12 months of age, the infant gut microbiota was dominated by *Bacteroides* (33.4%), *Veillonella* (9.5%), and *Bifidobacterium* (9.1%) (Figure 2). There were statistical differences between microbiota composition at 5 and 12 months; the abundance of *Faecalibacterium*, *Parasutterella*, *Bacteroides*, and *Akkermansia* significantly increased, and *Escherichia–Shigella*, *Enterobacteriaceae*, and *Bifidobacterium* decreased (*p* < 0.05).

Table 5 presents the correlation between breast milk components and the infant fecal microbiota. In this analysis, fat content in breastmilk negatively correlated with *Veillonella* abundance (*p* = 0.02), and oligosaccharides positively correlated with Lachnospiraceae (*p* = 0.03) at 5 months. Additionally, a tendency toward a negative correlation was found between fat content in breast milk and the abundance of Veillonellaceae (*p* = 0.055), and there was a tendency toward a positive correlation between lactose and Lachnospiraceae (*p* = 0.06).

**Figure 3:**
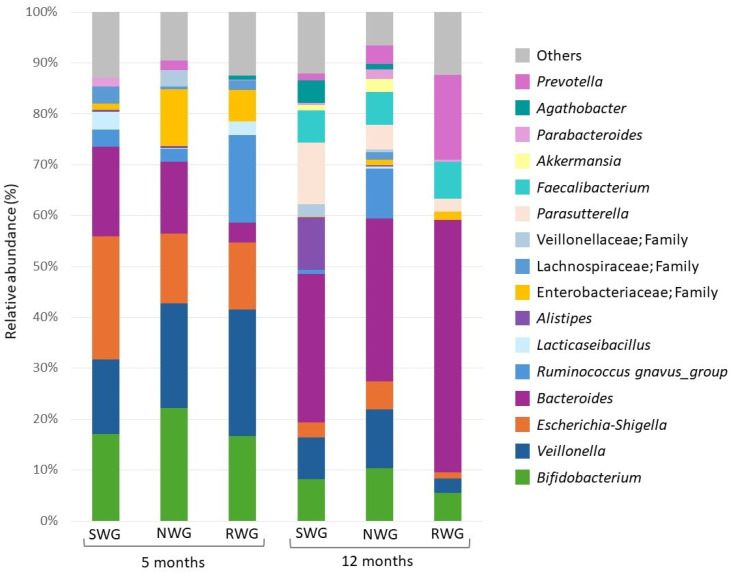
Infant gut microbiota at 5 and 12 months grouped by infant growth categories. NWG: normal weight gain; SWG: slow weight gain; RWG: rapid weight gain. The gut microbiota at 5 months was grouped according to the growth categories at period 1 (0 to 5.5 months), and the gut microbiota at 12 months was grouped according to the growth categories at period 2 (5.5 to 12 months). Period 1: SWG (n = 8), NWG (n = 13), and RWG (n = 5). Period 2: SWG (n = 7), NWG (n = 14), and RWG (n = 3).

There were some associations between the infant gut microbiota and infant growth. At period 1, infants with NWG had a higher abundance of the genus *Bifidobacterium* and the family Enterobacteriaceae at 5 months of age, although these differences did not reach statistical significance. Infants that presented RWG in period 1 had a higher abundance of *Ruminococcus gnavus* (*p* = 0.03), and infants who experienced SWG in period 2 had a higher abundance of *Alistipes* at 12 months of age (*p* = 0.01) (Figure 3).

## 4. Discussion

Looking for reasons to explain the different growth patterns of exclusively breastfed Mexican infants, we assessed the relationship between maternal diet, breast milk composition, infant gut microbiota, and weight gain in two periods: 1 (0 to 5.5 months) and 2 (5.5 to 12 months).

We found differences in the gut microbiota according to infant weight gain categories. In period 1, infants that experienced RWG had a higher abundance of *Ruminococcus gnavus*, a taxon found in the fecal microbiota of obese adults [26], and its abundance was positively associated with increases in weight-for-length Z-score (WLZ) measurements in Bangladeshi children [27]. *Ruminococcus gnavus* produces propionate and butyrate in the colon, which could play an important role in energy harvesting and affect weight gain [28,29]. Also, in period 1, in the infants who experienced NWG, there was a tendency toward a higher abundance of *Bifidobacterium* than in infants in the other two groups. *Bifidobacterium* is one of the most abundant genera in the gut microbiota of breastfed infants, given its preference for HMOs degradation, and while infants are exclusively breastfed, *Bifidobacterium* and other genera such as *Veillonella* dominate and preserve the stability of the gut microbiota and prevent precocious maturation of the infant microbiota, protecting against rapid weight gain [29].

In period 2, in RWG infants, there was a tendency toward a higher abundance of *Bacteroides* than SWG and NWG. In previous studies of children with type 1 diabetes at onset in the same population, we found a great increase in *Bacteroides* abundance [30]. A *Bacteroides* abundance has been associated with an adult-like gut microbiota which is associated with rapid weight gain [31]. The genus *Alistipes* was increased in infants experiencing SWG, and it was previously found in healthy children in this population [32]. Given that *Alistipes* was particularly high in one of the samples, it is not possible to suggest that its abundance slows weight gain or prevents RGW.

In general, the fecal microbiota at 5 months of age was dominated by the genus *Veillonella, Bifidobacterium,* and *Escherichia-Shigella*; previous studies have found that these genera dominate the gut microbiota of exclusively breastfed infants at 5 months of age [33]. At 12 months of age, the infant gut microbiota was dominated by the genus *Bacteroides*. This finding is in line with previous studies, where after the introduction of solid foods, the gut microbiota of infants shifted toward an adult-like microbiota [34].

*Veillonella* was highly abundant in the infant gut microbiota at 5 and 12 months of age. This genus is abundant in breast milk and it has been identified as one of the signature genera found in the gut microbiota of breastfed infants, contributing to a low oxygen concentration and promoting lactate metabolism [35,36]. The fact that *Veillonella* and *Bifidobacterium* were still dominant, after *Bacteroides,* in the fecal microbiota at 12 months of age speaks of the high prevalence of extended breastfeeding in the sample since 92% of the infants were still breastfed at 12 months of age. This extended breastfeeding may be one of the reasons we found a low prevalence of RWG (16%) at 12 months of age. In other studies, infants who were weaned before 12 months of age had a more mature microbiota than their breastfed counterparts, which is associated with rapid growth trajectories [29,36]. This points out the key role breastfeeding has in shaping the microbial communities in an infant’s gut and its role in the infant’s future health.

Breast milk is the source of nutrients for fermentation and proliferation of exclusively breastfed infant gut microbiota specimens. In our study, the HMOs content positively correlated with Lachnospiraceae at 5 months. *Ruminococcus gnavus* is one of the most abundant species from Lachnospiraceae in the gut microbiota of breastfed infants, and *R. gnavus* ferments HMOs; therefore, this relation can be explained by the preference this species has on HMO [28,37]. Additionally, the fat content in breastmilk negatively correlated with *Veillonella* abundance. This is in line with a previous study that reported the relation between breast milk fatty acids and the abundance of *Veillonella* in the infant gut microbiota [38].

Regarding breast milk composition, the maternal pre-gestational BMI was negatively correlated with protein concentration at 5 months postpartum. We hypothesize that protein was displaced by the high fat content in breast milk, which was 47.7 mg/mL. The fat ingestion of the mothers in our study contributed to 34.9% of the energy intake, and 52% of them were overweight/obese. Therefore, maternal BMI could indirectly influence protein concentration because of the high fat content, although we did not find this relation as in other studies [39]. Although we did not find a direct correlation between maternal diet and breast milk composition, the association could be indirect because the excessive caloric intake increases BMI, and this influences the breast milk composition.

Protein concentration in breast milk was related to infant growth, infants that experienced NWG at period 1 consumed more protein than those who experienced SWG, which agrees with a previous study [40]. This influence of protein on weight gain may be mediated by insulin-like growth factor 1, of which the secretion is increased by a high protein intake and, therefore, promotes infant growth [41]. Although we did not measure breast milk volume intake, the concentration of breast milk components is a proxy for the amount of nutrients infants receive and its potential influence on infant growth.

We found that 18.5% and 16% of infants experienced RWG in periods 1 and 2, respectively, which suggests that the slope of infant weight gain trajectories tends to decrease as infants age, in line with previous findings about infant weight gain over time [42]. To our knowledge there is only one study evaluating infant weight gain in the Mexican population. This study found that 33% of infants experienced RWG in the first year of life [4]. This is almost the two-fold the prevalence found in our study, and this difference could be due to differences in the assessment of infant weight gain or the duration of exclusive breastfeeding, which has been associated with the prevention of rapid weight gain [3].

In our study, breastfeeding shaped the gut microbiota of exclusively breastfed infants, and its structure was associated with infant weight gain trajectories. Our findings are aligned with other studies, such as one with Hispanic infants whose fecal microbiota diversity and richness at 1 month of age predicted rapid weight gain in the first year of life [31]. In Spanish infants, a lower abundance of *Bifidobacterium* was associated with weight gain in the first 18 months of life [43]. Therefore, we can propose that breastfeeding offers protection against rapid weight gain through infant gut microbiota modulation. Perhaps the microbiota structure was the reason that in the study by Leth-Møller et al., even the infants with rapid weight gain who were breastfed for at least 4 months did not experience childhood obesity [2].

One of the study’s strengths is the measurement of infant growth with conditional weight gain. Infant weight gain is regularly measured by the difference between the current Z-score and the previous one [17]. However, by measuring conditional weight gain, we are measuring the difference between the expected weight gain and the actual weight gain, which helps reduce the discrepancies that can be found when evaluating infant weight gain in different periods, and it can provide a proxy of the risk of obesity that exists in this population [16]. Additionally, most studies assessing infant weight gain use the weight-for-age Z-score (WAZ); however, there is evidence that the weight-for-length Z-score is a more accurate proxy for adiposity than WAZ [44].

One of the limitations of this study is that some perinatal factors, such as maternal pre-gestational weight, weight gained during pregnancy, and infant weight and length at birth, were reported by mothers; therefore, these data could be subject to bias. Also, data on complementary feeding were not collected, which would have helped interpret the infant gut microbiota composition at 12 months of age. Additionally, in this study, we found some tendencies that may have been significant if we had a larger sample size; therefore, there is a need for further research in this area with larger sample sizes. Also, the use of the snowball sampling method could have introduced selection bias. Lastly, the measurement of HMOs concentration by the indirect method could be a limitation. Including a more extensive analysis of the HMOs composition and fatty acids of breast milk would have provided a deeper understanding of the influence of breastfeeding on infant microbiota and growth.

## 5. Conclusions

In our study, breastfeeding shaped the gut microbiota of exclusively breastfed Mexican infants, and the gut microbiota structure was associated with infant weight gain trajectories. Given that breastfeeding influences gut microbiota composition and is linked to infant weight gain, promoting exclusive breastfeeding could serve as a preventive strategy against rapid weight gain and the risk of future obesity. Healthcare providers should emphasize the role of breastfeeding in shaping the infant microbiota to support infant health and optimal growth. Additionally, these insights reinforce the need for policies that encourage breastfeeding practices to reduce the risk of obesity and related metabolic disorders.

## Figures and Tables

**Table 1:** General and perinatal characteristics of mother–infant dyads.

Maternal Variable	n = 27
Age, years	32.3 ± 4.4
Weight gain during pregnancy, kg	11.5 ± 7.2
Weight at 5 months pp, kg	68.8 ± 15.5
Height, cm	162.3 ± 6.5
Pre-gestational BMI, kg/m^2^	25.5 ± 5.2
BMI at 5 months pp, kg/m^2^	26.0 ± 5.2
*Underweight*	1 (3.7)
*Normal weight*	12 (44.4)
*Overweight*	9 (33.3)
*Obese*	5 (18.5)
Primiparous	13 (48)
Vaginal delivery	15 (56)
**Infant Variable**	
Gender, female	14 (51.8)
Gestational age, weeks	38.9 ± 1.2
Weight at birth, kg	3.3 ± 0.4
Length at birth, cm	50.1 ± 1.8
Weight at 5.5 months, kg	7.5 ± 1.3
Length at 5.5 months, cm	65.4 ± 3.1
Weight at 12 months, kg *	9.5 ± 1.5
Length at 12 months, cm *	75.2 ± 3.5
WLZ at birth	−0.3 ± 1.1
WLZ at 5.5 months	0.3 ± 1.3
WLZ at 12 months *	0.1 ± 1.2
Fed breast milk and expressed milk	14 (51.8)
Breastfed at 12 months of age *	23 (92)
Age of introduction of solid foods, months *	6.04 ± 0.4

Values are the mean ± standard deviation or n (%). * n = 25. BMI: body mass index; pp: postpartum; WLZ: weight-for-length Z-score.

**Table 2:** Breast milk composition at 5 months postpartum.

Component	Mean ± SD
Protein (mg/mL)	14.2 ± 4.6
Lipids (mg/mL)	47.7 ± 15.2
Lactose (mg/mL)	64.6 ± 11.7
HMOs (mg/mL)	14.8 ± 9.4

n = 27. HMOs: human milk oligosaccharides; SD: standard deviation.

**Table 3:** Pearson correlation between maternal body mass index and breast milk composition at 5 months postpartum.

Component	Maternal BMI			
	Pre-Gestational	*p* Value	At 5 Months pp	*p* Value
Protein (mg/mL)	−0.42	0.02	−0.38	0.05
Lipids (mg/mL)	−0.19	0.35	−0.22	0.26
Lactose (mg/mL)	0.26	0.19	0.20	0.30
HMOs (mg/mL)	−0.34	0.09	−0.33	0.10

n = 27. BMI: body mass index; HMOs: human milk oligosaccharides; pp: postpartum.

**Table 4:** Differences in breast milk composition at 5 months postpartum by infant growth categories in period 1 (0 to 5.5 months).

Breast Milk Component	Infant Growth 0–5.5 Months		
	SWG (n = 8)	NWG (n = 14)	RWG (n = 5)
Protein (mg/mL)	10.7 ± 3.9 ^a^	16.6 ± 4.3 ^b^	12.9 ± 1.1 ^ab^
Lipids (mg/mL)	46.9 ± 14.6 ^a^	49.1 ± 17 ^a^	44.9 ± 12.6 ^a^
Lactose (mg/mL)	64.8 ± 17.4 ^a^	64.1 ± 9.7 ^a^	65.5 ± 7.3 ^a^
HMOs (mg/mL)	18.0 ± 12.1 ^a^	15.1 ± 8.9 ^a^	9.4 ± 3.9 ^a^

HMOs: human milk oligosaccharides; NWG: normal weight gain; SWG: slow weight gain; RWG: rapid weight gain. Different superscripts for categories of infant growth in each breast milk component denotes significant differences between groups. ^ab^ denotes that a breast milk component in a category is not different than in category ^a^ and in category ^b^. The analysis was based on an ANOVA using the Bonferroni test, *p* < 0.05.

**Table 5:** Pearson correlation between breast milk components at 5 months postpartum and the infant fecal microbiota at 5 months of age.

Taxa Relative Abundance	Breast Milk Component (mg/mL)			
	Lipids	Proteins	Lactose	HMOs
*Ruminococcus gnavus group*	0.25 (0.44)	−0.04 (0.89)	−0.23 (0.49)	0.32 (0.36)
*Bifidobacterium*	0.32 (0.12)	0.18 (0.39)	0.18 (0.38)	−0.21 (0.34)
*Escherichia-Shigella*	0.22 (0.31)	−0.04 (0.87)	0.01 (0.97)	0.26 (0.29)
*Veillonella*	−0.50 (0.02)	−0.33 (0.16)	0.23 (0.33)	−0.18 (0.45)
*Bacteroides*	0.23 (0.44)	0.15 (0.61)	0.08 (0.77)	−0.19 (0.55)
Enterobacteriaceae	−0.16 (0.64)	0.21 (0.51)	0.28 (0.39)	−0.48 (0.13)
Veillonellaceae	−0.45 (0.055)	−0.29 (0.21)	0.19 (0.41)	−0.18 (0.46)
Lachnospiraceae	0.27 (0.46)	−0.41 (0.70)	0.63 (0.06)	−0.75 (0.03)

Data are presented as correlation coefficient (*p* value). HMOs: human milk oligosaccharides. The abundances of fecal microbiota taxa were transformed by natural logarithm to achieve normal distribution.

## Data Availability

The data presented in this study are available upon request to the corresponding author.

## Supplementary Materials

The following supporting information can be downloaded at: https://www.mdpi.com/article/10.3390/nu17050826/s1, Figure S1: Infant fecal microbial diversity at 5 and 12 months of age grouped by infant growth categories.
