# Supplementary material for: Breastfeeding Shapes the Gut Microbiota and Its Structure Is Associated with Weight Gain Trajectories in Mexican Infants
Source: Nutrients. 2025 Feb 27;17(5):826. doi: 10.3390/nu17050826 (PMC11901506; doi:10.3390/nu17050826)
Supplement: Supplementary file 1 [file nutrients-17-00826-s001.zip › nutrients-3492592-supplementary.pdf]

Supplementary material

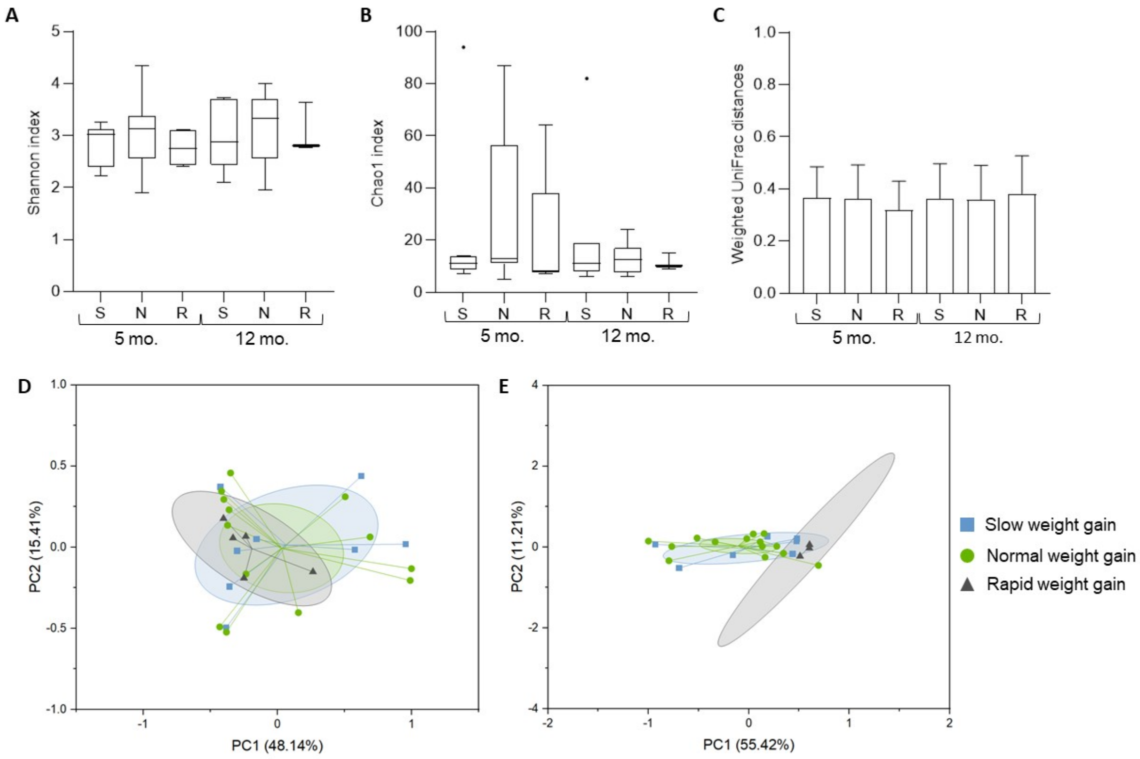

Figure S1. Infant fecal microbial diversity at 5 and 12 months of age grouped by infant growth categories. A. Shannon Index. B, Chao Index. C. Weighted UniFrac distance. D. Bray-Curtis dissimilarities at 5 months of age. E. Bray-Curtis dissimilarities at 12 months of age. N: normal weight gain; S: slow weight gain; R: rapid weight gain.The gut microbiota at 5.5 months was grouped according to the growth categories at period 1 (0 to 5.5 moths), and the gut microbiota at 12 months was grouped according to the growth categories at period 2 (5.5 to12 moths). Period 1: S (n=8), N (n=13) and R (n=5). Period 2: S (n=7), N (n=14) and R (n=3).
